# A novel approach for gingiva thickness measurements around lower anterior teeth by means of dental magnetic resonance imaging

**DOI:** 10.1007/s00784-023-05459-4

**Published:** 2023-12-23

**Authors:** Linda Schwarz, Ewald Unger, André Gahleitner, Xiaohui Rausch-Fan, Erwin Jonke

**Affiliations:** 1 Division of Orthodontics, University Clinic of Dentistry, Medical University of Vienna, Sensengasse 2a, 1090 Vienna, Austria; 2 Center for Medical Physics and Biomedical Engineering, Medical University of Vienna, Spitalgasse 23, 1090 Vienna, Austria; 3 Department of Biomedical Imaging and Image-Guided Therapy, Medical University of Vienna, Spitalgasse 23, 1090 Vienna, Austria; 4 Center of Clinical Research, University Clinic of Dentistry, Medical University of Vienna, Sensengasse 2a, 1090 Vienna, Austria

**Keywords:** MRI, Lower incisors, Gingival thickness, Orthodontic treatment, Gingival phenotype

## Abstract

**Objective:**

This diagnostic accuracy study aims to present the first measurements of gingiva thickness around lower anterior teeth using dental magnetic resonance imaging (MRI) and to compare these measurements with two established methods: (1) gingival phenotype assessment via periodontal probing, and (2) the superimposition of cone-beam computed tomography (CBCT) scans with intraoral scans of teeth and gums.

**Materials and methods:**

Ten patients with substantial orthodontic treatment need and anterior mandibular crowding were consecutively included in this clinical case series. After periodontal probing, each patient underwent a CBCT scan, an intraoral scan of the mandible, and an MRI investigation using a novel mandibula 15-channel dental coil.

**Results:**

The mean gingiva thickness was 0.72 mm measured on MRI and 0.97 mm measured on CBCT, with a mean difference between the measurement methods of 0.17 ± 0.27 mm (*p* < 0.001). Measurement agreement between the index tests (MRI and CBCT) and the clinical reference standard (probing) yielded an overall percent agreement of 64.94% and 47.02% for MRI and CBCT, respectively. Teeth with thin phenotypes were associated with lower soft tissue dimensions in both free (MRI: 0.56 mm vs. CBCT: 0.79 mm) and supracrestal gingiva (MRI: 0.75 mm vs. CBCT: 1.03 mm) when compared to those with thick phenotypes. However, only the measurements obtained from MRI scans showed statistically significant differences between the two phenotypes.

**Conclusion:**

Dental MRI successfully visualizes delicate structures like the gingiva in the anterior mandible and achieves a high correlation with superimposed CBCT scans, with clinically acceptable deviations.

**Clinical relevance:**

The present study helps to establish dental MRI as a radiation-free alternative to conventional radiographic methods.

## Introduction

Understanding the role of gingiva thickness has increasingly gained the attention of researches in recent years, marking a notable shift from the focus on gingiva width, which has already been the subject of numerous investigations [1]. The characterization of gingiva morphology has introduced two main types: scalloped and thin or flat and thick gingiva [2]. In the event of inflammation or any other type of insult, the soft tissue in a thin phenotype responds with more inflammatory changes and an increased likelihood of gingival recession [3]. In the past, approaches to measuring gingiva thickness encompassed invasive methods such as transgingival insertion of an endodontic instrument [4, 5], a syringe [6], or caliper measurements after tooth extraction [7, 8] under local anesthesia. A minimally invasive method of visual inspection of the transparency of a periodontal probe through the sulcus emerged as an alternative, yielding satisfactory accuracy [7, 9]. However, this method’s outcomes do not directly measure gingival thickness; they rather yield a dichotomous result that is susceptible to inter-observer and -operator variations [10].

Orthodontic treatment can inadvertently lead to dehiscence in the buccal bone plate due to unwanted proclination of the lower incisors in the course of initial orthodontic treatment [11]. This phenomenon is particularly pronounced in patients with thin gingiva phenotype [12]. This, in turn, increases the risk for gingival recessions, as they are normally preceded by inadequate alveolar bone support for the affected tooth. Lower incisors were found to be the most susceptible teeth to develop labial recessions [13]. Consequently, knowledge of both soft and hard tissue dimensions before onset of orthodontic treatment may therefore help to devise the best possible orthodontic treatment plan. Nonetheless, the impact of orthodontic treatment on the emergence of gingival recessions remains a subject of ongoing debate within the literature [13–15]. This might stem from the lack of prospective trials investigating tissue dimensions around the teeth during orthodontic treatment [13, 14], along with the uncertainty about diagnostic reliability of clinically relevant examinations [15].

Cone-beam computed tomography (CBCT) has emerged as a dependable tool for assessing hard tissue dimensions [16, 17]. However, the direct measurement of soft tissues presents challenges due to their adhesion to lips, cheeks, and tongue [18, 19], in addition to the CBCT’s limitations in terms of resolution and contrast [20]. Recent research attempts have explored innovative techniques, including the use of lip retractors [21] and the superimposition of CBCT images onto intraoral scans to assess labial gingiva thickness [22, 23]. Nevertheless, a comprehensive evaluation of mandibular periodontal conditions utilizing this approach has not yet been undertaken [21, 23, 24]. Moreover, CBCT may not be ideal for follow-up examinations, especially in adolescents, due to concerns regarding radiation exposure. In contrast, magnetic resonance imaging (MRI) examinations provide excellent contrast for soft tissues; however, it lacks in capability to capture hard tissue signals [25]. Literature has already introduced the successful in vivo application of a dedicated dental coil for MRI applications [26–28]. Through the utilization of such coils and the optimization of MRI sequencing [29], the achievement of high resolution imaging for both soft and hard tissues becomes possible.

The primary objective of this consecutive controlled case series was to assess the potential of dental MRI for measurement of gingiva dimensions around the lower anterior teeth in patients with anterior crowding, and who are about to start orthodontic treatment. Furthermore, the study aimed to compare these measurements with those derived from superimposed CBCT scans and from periodontal probing.

## Materials and methods

### Patients

The sample consisted of adolescent patients with substantial orthodontic treatment need and anterior mandibular crowding who were consecutively included in this prospective clinical case series. They were all about to undergo orthodontic therapy at the Department of Orthodontics of the University Clinic of Dentistry (Medical University of Vienna, Austria) between April 2022 and April 2023. The following inclusion criteria had to be met: 12–18 years of age, substantial orthodontic treatment need according to the Index of Orthodontic Treatment Need (IOTN) [30], anterior crowding in the mandible of > 3 mm, and fully erupted permanent teeth in the lower jaw. Exclusion criteria were history of claustrophobia, presence of gingival recessions, cranio-maxillofacial anomalies, and medication intake that influence the structure of periodontal structures. The patients and caregivers gave written informed consent for the MRI examinations and for publication of this case series, including accompanying images. The study protocol was was conducted in accordance with the Declaration of Helsinki and was approved by the Ethics Committee of the Medical University of Vienna (EK Nr: 1654/2021).

### Study protocol

The patients were scheduled for 3D imaging of the mandible using MRI, and CBCT at the Department of Radiology, University Clinic of Dentistry, Vienna, before application of the orthodontic appliance in the lower jaw. As a clinical reference test, visual inspection of the transparency of a periodontal probe (Marquis probe) through the sulcus was performed to assess the gingival phenotype [9]. Visibility of the probe (visible: thin phenotype or invisible: thick phenotype) was recorded. Finally, an intraoral scan of the mandible was conducted in the same appointment using an intraoral scanner (iTero 2, Align Technology, California).

### MRI and CBCT acquisition

**Fig. 1:**
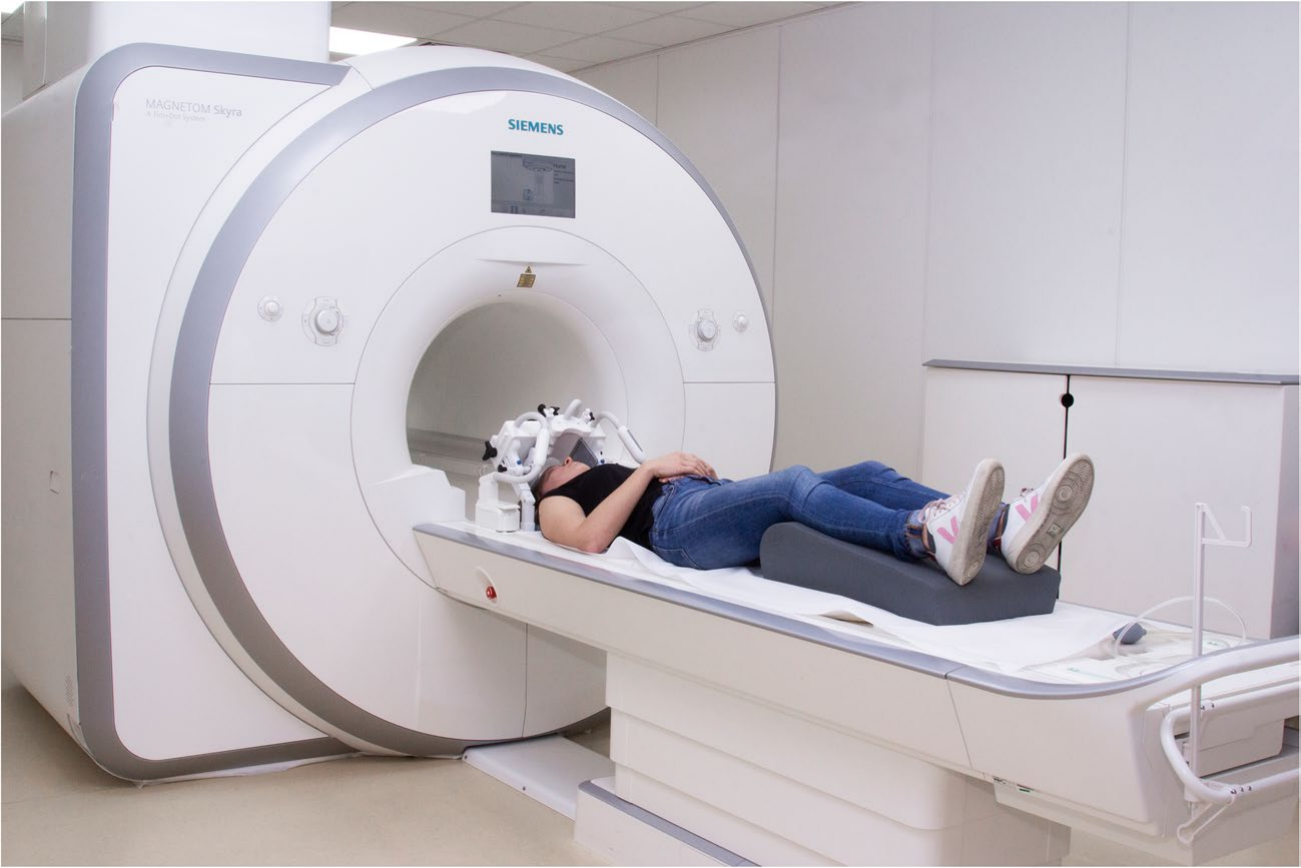
Example of a patient right before image acquisition. The dental mandibular coil is not yet fully adjusted around the patient’s head

MRI examinations were performed using a 3 T MRI system (Magnetom Skyra, Siemens Healthcare GmbH, Germany) and a Mandibula 15-Ch Dental Coil (Noras MRI products Gmbh, Germany). The patients were examined with lips and tongue in a resting position. A cotton gauze was inserted in the anterior region of the lower vestibule without pressure for retraction of the lower lip. A PD weighted sequence with fat suppression was applied. Sequence parameters were: 2D sequence, 150 mm FoV read, 142 degrees flip angle, 46 sections, time of acquisition 5:58 min, base resolution 320, and low SAR RF pulse type (Fig. 1).

CBCT examinations were performed using a 3D Accuitomo imaging system (3D Accuitomo, J.Morita Corporation, Japan). The volume was restricted to 40 × 40 mm, and the settings were as follows: 90 kV, 7 mA, scan time 10.5 s, voxel size 0.8/0.8/0.8 mm.

### MRI and CBCT measurements

**Fig. 2:**
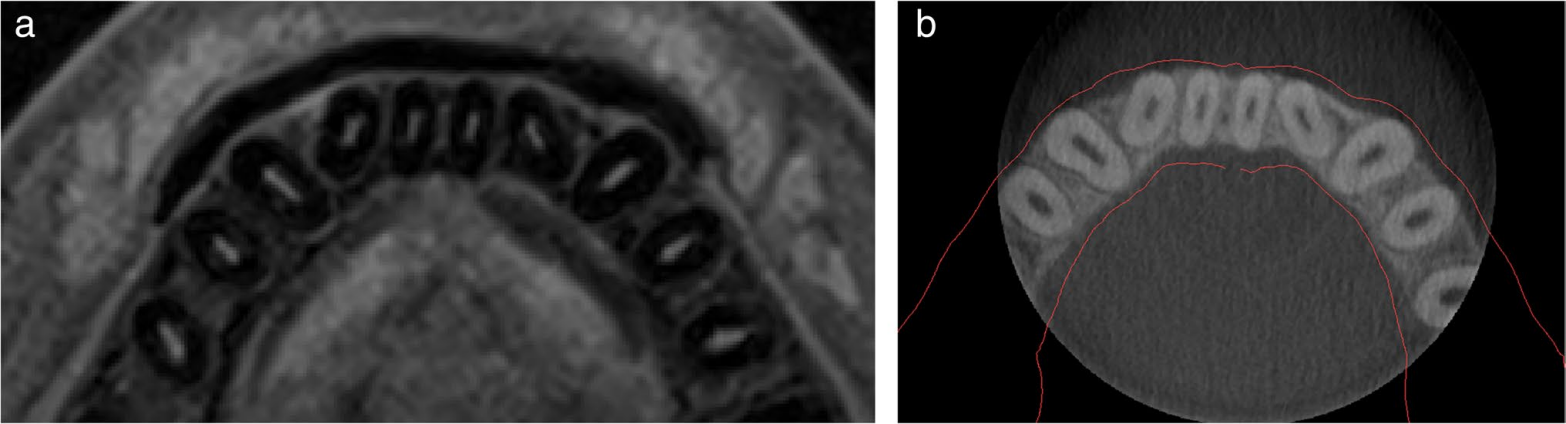
Example of analyzed image datasets: **a** horizontal plane of MRI; **b** horizontal plane of superimposed CBCT/intraoral scan of the same patient

**Fig. 3:**
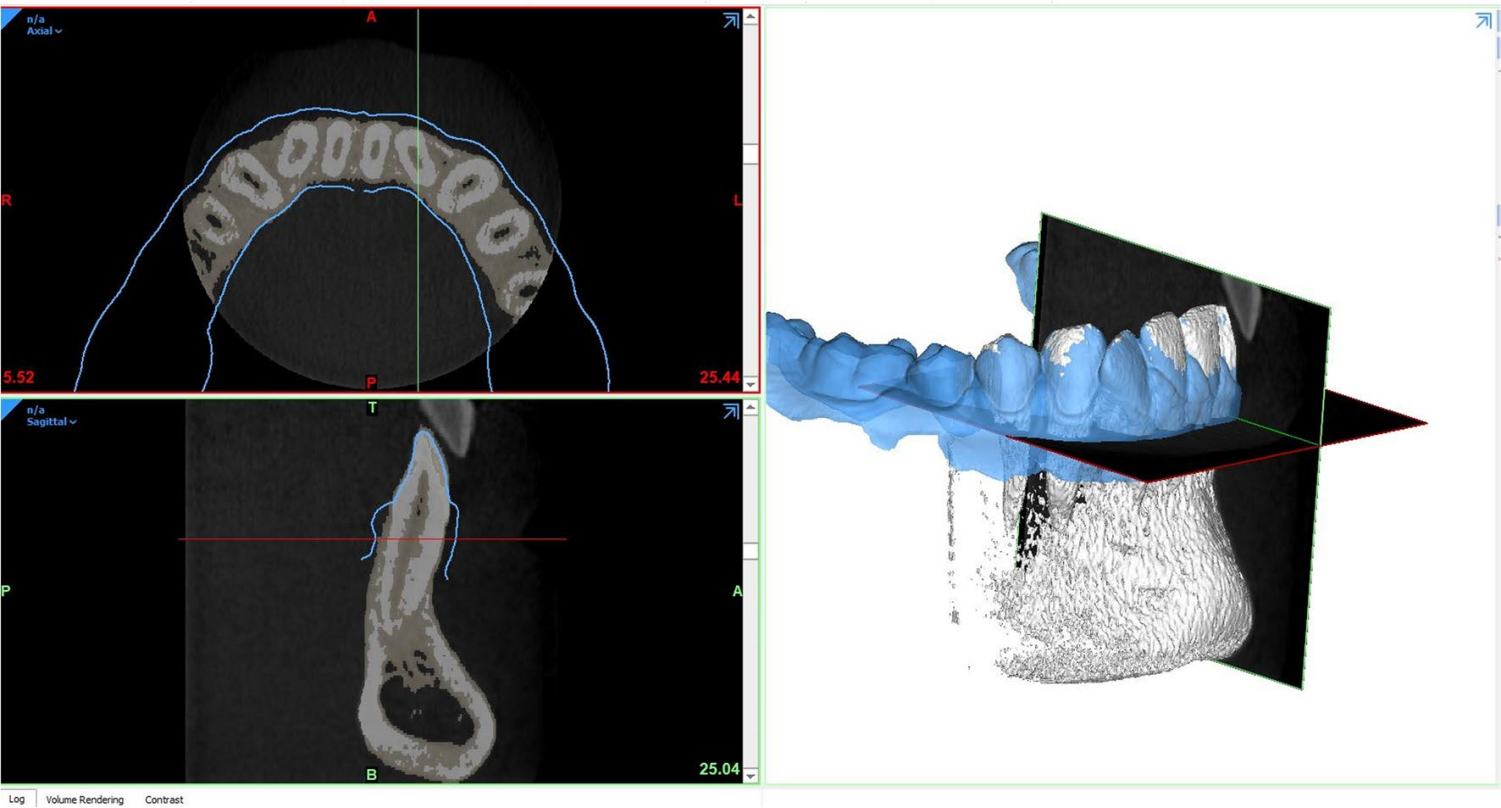
Representative example of the superimposition of an intraoral scan (blue color) with the CBCT dataset in Materialize Mimics after the creation of a 3D reconstruction from the DICOM data

After clinical assessment of periodontal phenotypes by probing, the two index tests were conducted: the thickness of gingiva at different apico-coronal levels was measured on superimposed CBCT and MRI scans to establish mean values for lower incisors in thick and thin gingival phenotypes. Image reconstruction of both MRI and CBCT scans for visual analysis was performed using Mimics Innovation Suite (Materialise, Belgium). All image reconstruction steps and all the measurements were done by the same examinator (L.S.). To allow for measurements of soft tissues on CBCT scans, the DICOM-data of the radiographic examination was superimposed with the stereolithography (STL-)file of the corresponding intraoral scan by using Mimics Innovation Suite (Fig. 2b). For superimposition, the hard tissues in the CBCT scan (i.e., bones, teeth) were reconstructed using the segmentation tools in Mimics. This segmentation process was achieved by creating a “mask” from the DICOM data using the CBCT threshold setting for enamel suggested by the software. By doing this, a 3D visualization of the CBCT dataset was created (Fig. 3), and the 3D model of the intraoral scan was manually moved to align with the mask. Then, detailed aligning was achieved using the global alignment tool of the software.

**Fig. 4:**
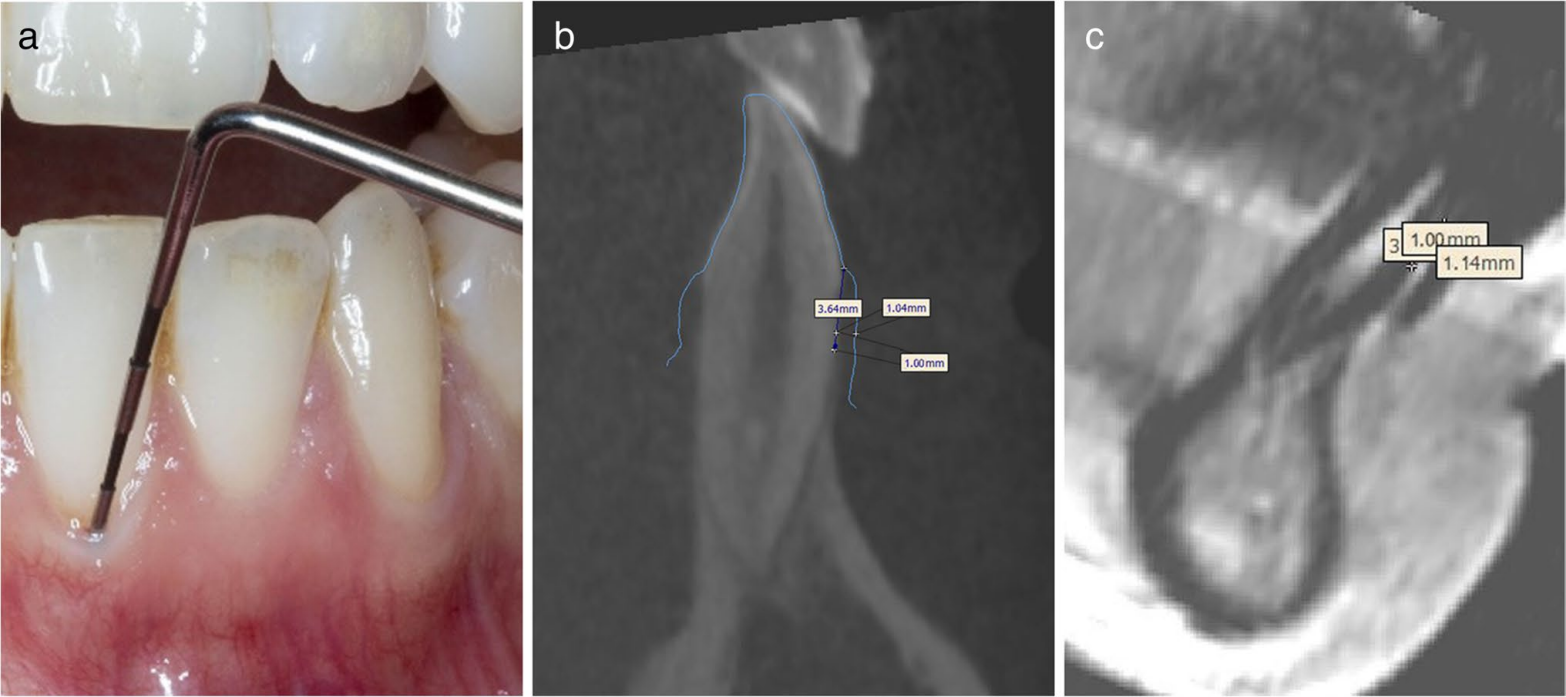
Representative examples of the three measurement methods: **a** periodontal probing of the gingival sulcus to assess probe transparency, **b** sagittal section through the axis of the lower left incisor with supracrestal measurement of the gingival thickness, **c** sagittal section through axis of the lower left incisor with supracrestal measurement of the gingival thickness

Axial, coronal, and sagittal planes were rotated to match the inclination, angulation, and rotation of the anterior teeth. Measurements were taken perpendicularly to the long axis of the tooth (Fig. 4).

The following parameters were chosen for measurement:

- free gingiva (1 mm below gingival margin)
- supracrestal gingiva (1 mm above alveolar crest)
- crestal gingiva (1 mm below alveolar crest)

Six dentogingival units (canine – canine) per MRI were examined, making a total of 18 measurements per MRI/CBCT. To assess intra-rater reliability, five MRI scans were measured twice in a random order 2 months after the first measurements by the same examiner (L.S.).

### Statistical analysis

Descriptive statistics were used to present soft tissue dimensions. All data were analyzed using SPSS version 25.0 (IBM Corporation, Armonk, NY). An evaluation of data distribution was conducted by visually inspecting histograms and applying the Kolmogorov–Smirnov-test. Pairwise *t* tests were performed to compare the differences between measurements obtained from MRI and CBCT scans, and Mann–Whitney *U* testing was performed to compare gingival dimensions between thick and thin phenotypes. For subgroup analysis, a log-transformation approach was employed to achieve a normal distribution of the data. Reported *p* values refer to *t* tests performed post log-transformation, whereas mean values and standard deviations were back-transformed to facilitate more comprehensive interpretation. For subgroup analysis of tooth types (canine, lateral incisor, and central incisor), mean values for left and right teeth were calculated. Intra-rater reliability was estimated through the computation of the intraclass correlation coefficient (ICC), which was applied to evaluate the thickness measurements of free, supracrestal, and subcrestal gingiva. Statistical analysis for measurement agreements between the index tests (MRI and superimposed CBCT) and the clinical reference standard (periodontal probing) was performed by calculating Overall Percent Agreement (OPA, overall agreement between both index tests and the reference), Negative Percent Agreement (NPA, agreement between thick phenotypes), and Positive Percent Agreement (PPA, agreement between thin phenotypes).The cutoff value of 0.8 mm was chosen to distinguish between thin and thick phenotypes on MRI and CBCT measurements.

## Results

Ten patients (five male, five female) with a mean age of 14.21 ± 1.82 years were included. Forty-three out of 60 teeth were classified as thin gingival phenotype via periodontal probing. Due to movement artifacts, one MRI dataset could not be used for further analysis, and the dataset had to be excluded. In total, 144 measurements from nine patients on MRI and 151 measurements on superimposed CBCT scans were taken (Table 1). ICC yielded a good intra-class correlation of 0.837 of average measures (95% CI: 0.752–0.892).

**Table 1:** Case summary

		Teeth	Measurements (missing values)^1^
MRI	Central incisor	18	50 (4)
	Lateral incisor	18	49 (5)
	Canine	18	45 (9)
CBCT	Central incisor	18	53 (1)
	Lateral incisor	18	52 (2)
	Canine	17	46 (8)

^1^Cases were considered missing values when the tissue borders were not clearly visible

### Difference between MRI and CBCT measurements

Measurements obtained from superimposed CBCT scans consistently reached higher values when compared to corresponding measurements obtained from MRI, as demonstrated in Tables 2 and 3. As depicted in Table 2, the thickness of the gingiva was lowest in the free gingiva region (0.62 mm on MRI and 0.85 mm on CBCT, *p* < 0.001), while the highest mean value was observed in the supracrestal gingiva (0.85 mm on MRI and 1.10 mm on CBCT, *p* < 0.001). The mean difference between measurements taken from MRI and superimposed CBCT scans accounted for 0.17 ± 0.27 mm and was statistically significant (Table 2, *p* < 0.001). Within the free gingiva region, measurements around each of the three tooth types (central incisor, lateral incisor, and canine) exhibited significant differences between MRI and CBCT (Table 3). The mean differences in free gingiva thickness measurements were 0.16 mm for central incisors (*p* < 0.001), 0.20 mm for lateral incisors (*p* = 0.028), and 0.15 mm around canines (*p* = 0.042). The most pronounced difference in the supracrestal gingiva was observed around lateral incisors with a difference of 0.24 mm (*p* = 0.024). Overall, both the highest and smallest differences, while lacking statistical significance, were measured in the subcrestal region, accounting for 0.087 mm around lateral incisors (*p* = 0.333) and for 0.26 mm around canines (*p* = 0.155), as presented in Table 3.

**Table 2:** Mean thickness (mm) of gingiva measured at different apico-coronal levels on MRI and superimposed CBCT

	MRI (N)	CBCT (N)	Mean Difference° (N)	*p* value
Free gingiva	0.62 ± 0.65 (54)	0.85 ± 0.32 (53)	0.17 ± 0.21 (53)	< 0.001*
Supracrestal gingiva	0.85 ± 0.67 (47)	1.10 ± 0.33 (52)	0.19 ± 0.30 (47)	< 0.001*
Subcrestal gingiva	0.73 ± 0.73 (43)	0.95 ± 0.29 (46)	0.14 ± 0.29 (39)	0.005*
All	0.72 ± 0.67 (144)	0.97 ± 0.29 (151)	0.17 ± 0.27 (139)	< 0.001*

° “Difference” refers to the mean difference in gingiva thickness between corresponding measurements obtained from superimposed CBCT scans and MRI scans. Positive values indicate a greater thickness measured on CBCT, while negative values indicate a greater thickness measured on MRI

*p values < 0.05 are considered statistically significant

**Table 3:** Mean difference of gingiva thickness measurements (mm) obtained from superimposed CBCT scans and MRI scans depending on region and tooth type

Region	Tooth type (N)	Mean difference°	*p* value
Free gingiva	Central incisor (18)	0.16 ± 0.16	< 0.001*
	Lateral incisor (18)	0.20 ± 0.26	0.028*
	Canine (17)	0.15 ± 0.20	0.042*
Supracrestal gingiva	Central incisor (16)	0.16 ± 0.32	0.219
	Lateral incisor (16)	0.24 ± 26	0.024*
	Canine (14)	0.20 ± 0.35	0.224
Subcrestal gingiva	Central incisor (16)	0.12 ± 0.26	0.172
	Lateral incisor (14)	0.087 ± 0.32	0.333
	Canine (9)	0.26 ± 0.30	0.155

Bonferroni-Holm correction was applied for multiple testing

° “Difference” refers to the mean difference in gingiva thickness between corresponding measurements obtained from superimposed CBCT scans and MRI scans. Positive values indicate a greater thickness measured on CBCT, while negative values indicate a greater thickness measured on MRI

*p values < 0.05 are considered statistically significant

**Fig. 5:**
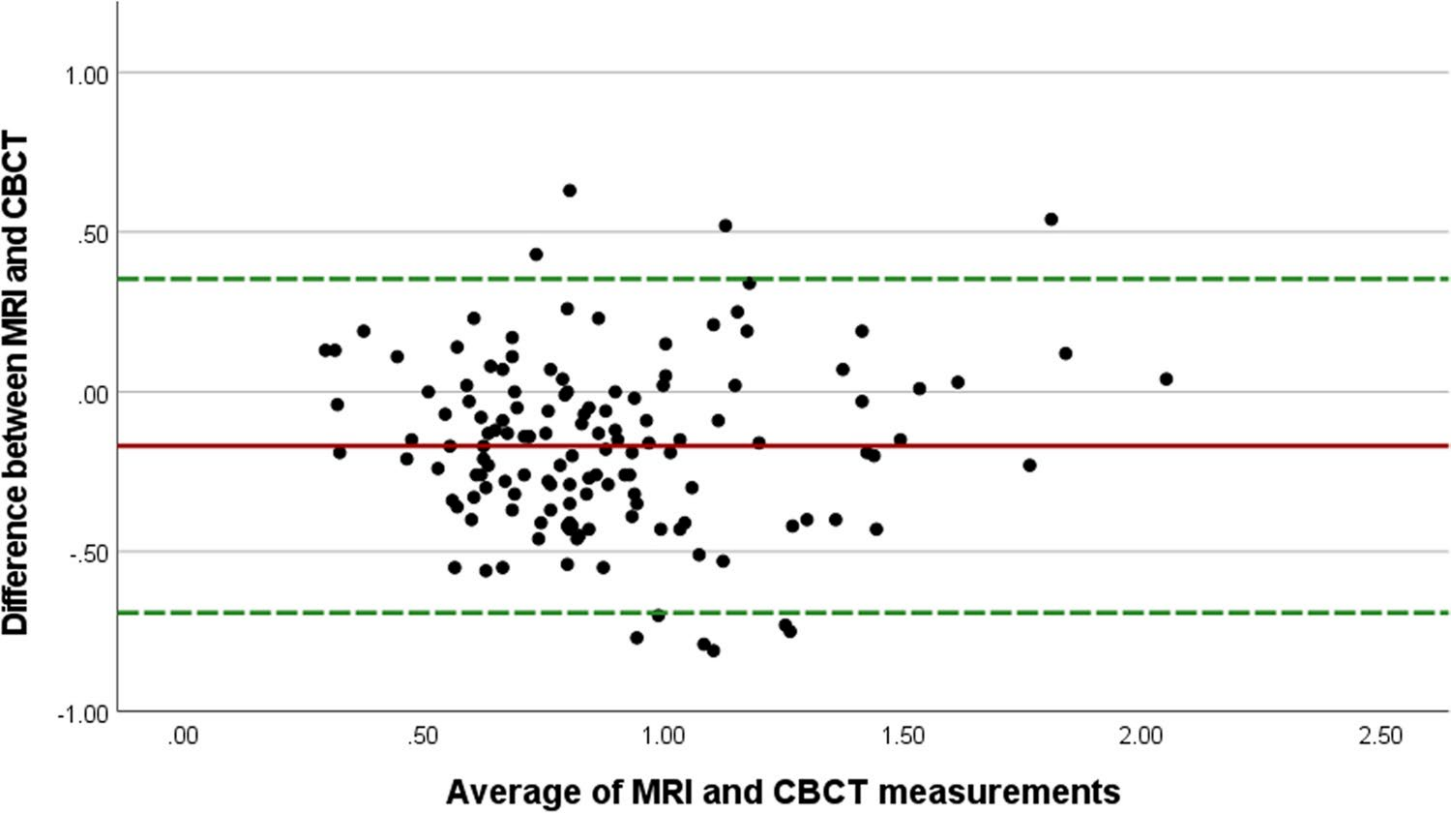
Agreement between MRI and CBCT measurements (Bland–Altman plot)

The Bland–Altman plot (Fig. 5) showed that the mean bias between MRI and CBCT measurements was − 0.17 ± 0.27 mm, and the limits of agreement were − 0.69 and 0.35. This indicates good agreement between the two methods of measurement, but with a trend towards greater dimensions on CBCT measurements.

### Thin vs. thick phenotypes

Thin and thick gingival phenotypes, as assessed by periodontal probing, demonstrated a correlation with the corresponding measurements of the free and supracrestal gingiva obtained from MRI scans (as shown in Table 4). For teeth, where periodontal probing indicated thin gingival phenotypes (meaning the periodontal probe was visible through the periodontal sulcus), a statistically significant difference in gingival dimensions was seen in comparison to teeth with thick phenotypes. Specifically, within the free gingiva region, thin phenotypes exhibited a mean gingiva thickness of 0.56 mm, while thick phenotypes displayed a thickness of 0.82 mm (*p* = 0.030). In the supracrestal gingiva region, thin phenotypes had a mean gingiva thickness of 0.75 mm, whereas thick phenotypes exhibited a thickness of 1.11 mm (*p* = 0.012). However, no statistically significant association was observed between measurements obtained from superimposed CBCT scans and the phenotypes assessed through periodontal probing. Specifically, thin gingival phenotypes exhibited a free gingiva thickness of 0.79 mm on CBCT scans, while thick phenotypes displayed a thickness of 1.02 mm (*p* = 0.14). Likewise, in the region of the supracrestal gingiva, thin phenotypes showed a thickness of 1.03 mm, whereas thick phenotypes had a thickness of 1.26 mm (*p* = 0.141). Furthermore, dimensions of the subgingival gingiva did not demonstrate a significant correlation with gingival phenotypes, both in the MRI and superimposed CBCT scans.

**Table 4:** Gingiva thickness measurements (mm) obtained from superimposed CBCT scans and MRI scans in thin and thick gingival phenotypes

Region	Imaging method	Thin gingival phenotype (N)	Thick gingival phenotype (N)	*p* value
Free gingiva	MRI	0.56 ± 0.69 (39)	0.82 ± 0.65 (15)	0.030*
	CBCT	0.79 ± 0.26 (38)	1.02 ± 0.42 (15)	0.14
Supracrestal gingiva	MRI	0.75 ± 0.70 (33)	1.11 ± 0.69 (14)	0.012*
	CBCT	1.03 ± 0.27 (37)	1.26 ± 0.41 (15)	0.141
Subcrestal gingiva	MRI	0.78 ± 0.25 (32)	0.69 ± 0.72 (10)	0.868
	CBCT	0.93 ± 0.29 (34)	0.99 ± 0.31 (12)	0.540

Mann–Whitney *U* testing for differences in gingiva thickness between thin and thick phenotypes

Bonferroni-Holm correction was applied for multiple testing

*p values < 0.05 are considered statistically significant

The calculation of measurement agreement (Table 5) showed a higher overall agreement between MRI measurements and periodontal probing (OPA 64.94%) compared to CBCT measurements (47.02%). Measurement agreement between periodontal probing and CBCT or MRI measurements varied considerably depending on the region of measurement. In the free gingiva, MRI measurements had high agreement with periodontal probing for the detection of thin gingival phenotypes (PPA 87.18%), but only 46.67% agreement for the detection of thick phenotypes. In contrast, the PPA between MRI and probing was 74.36%, and the NPA was 73.33% in the supracrestal region.

**Table 5:** Measurement agreement between the index tests (MRI and CBCT) and the clinical reference standard (probing)

Region		OPA	PPA°	NPA
Free gingiva	MRI	75.93%	87.18%	46.67%
	CBCT	61.11%	58.97%	66.67%
Supracrestal gingiva	MRI	74.07%	74.36%	73.33%
	CBCT	37.03%	20.51%	80.00%
Subcrestal gingiva	MRI	48.15%	61.54%	13.33%
	CBCT	48.15%	46.15%	53.33%
Overall	MRI	64.94%	72.73%	71.43%
	CBCT	47.02%	45.45%	37.61%

*OPA* overall percent agreement, *PPA* positive percent agreement, *NPA* negative percent agreement

°PPA describes the measurement agreement for thin phenotypes, and NPA the measurement agreement for thick phenotypes

## Discussion

Gingival thickness directly impacts the biomechanical response of periodontal tissues to orthodontic forces. Thicker gingiva is often associated with increased tissue resilience, offering enhanced protection against the potential adverse effects of orthodontic forces [3]. In contrast, thin gingiva may be more susceptible to damage, leading to complications such as recession, root resorption, and compromised stability of teeth [31]. Precise assessment of gingival thickness helps orthodontists to choose optimal treatment modalities, force magnitudes, and appliances, thereby optimizing treatment outcomes while minimizing the risk of undesirable consequences. In clinical research, accurate knowledge of the dimensions of healthy gingival is essential for evaluating the results of periodontal surgery.

In the present study, the utilization of dental MRI exhibited an advantage in discriminating gingival phenotypes in comparison to CBCT, which is likely attributed to the good soft tissue contrast provided by MRI. Periodontal probing indicated a thin gingival phenotype in cases where the periodontal probe was visible through the periodontal sulcus. A statistically significant variation in gingival dimensions was noted between thin and thick phenotypes on measurements obtained from MRI scans, which was most prominently observed within the free gingiva and supracrestal gingiva. However, prior investigations primarily confirmed this correlation solely for free gingiva thickness [22, 23].

No universal cutoff values have yet been established to differentiate between thick and thin phenotypes [32]. Some studies define thick or thin phenotypes with gingival thickness threshold of 1 mm [5, 7], others with a threshold of 1.5 mm [33]. Differences in tissue thickness at different apico-coronal levels and the lack of consensus concerning the reference anatomical landmark may contribute to the inconsistencies observed in previous studies when defining gingiva phenotypes [22, 23, 34]. A study, which correlated thin and thick phenotypes with direct transgingival measurements, noted a mean gingiva thickness of 1.23 mm in thin phenotypes and of 1.73 mm in thick phenotypes [33], which does not correspond to the dimensions obtained in the present study (thin phenotypes with 0.75 mm vs. thick phenotypes with 1.11 mm in supracrestal gingiva). It is important to note that the mentioned study conducted the measurements halfway between the gingival margin and the mucogingival junction. This reference point cannot be directly correlated to the reference points adopted in the current study. Furthermore, the mean values acquired from the referenced study describe the thickness around both upper and lower incisors. However, when indirect assessment of gingival thickness (i.e., probing) was compared with direct measurements (radiographic or transgingival probing), studies found that the gingiva becomes non-transparent at a thickness of 0.8 mm [35–38]. In thin phenotypes, free gingival thickness was 0.65 mm [23] and 0.4 mm in the crestal region [38]. Therefore, in the present study, the threshold value of 0.8 mm was chosen as the cutoff value for the classification of thin and thick phenotypes.

Interestingly, the comparison of two different measurement techniques (transgingival probing vs. CBCT scans) revealed elevated tissue dimensions on CBCT images that are analogous to the current investigation [37]. Specifically, the mean gingiva thickness obtained through transgingival probing was 0.85 mm, while the corresponding value from CBCT scans was 0.94 mm. These measurements were taken at the level of the CEJ, which aligns closely with the supracrestal measurements adopted in the current study. Accordingly, the current study revealed a thickness of 0.85 mm on MRI and of 1.10 mm on CBCT images at the supracrestal level.

Clinical comparability between dental MRI and CBCT was demonstrated in earlier studies, even though CBCT had significantly heightened accuracy [39]. Remarkably, the relatively lower spatial resolution did not substantially impair measurement reliability [40]. However, some measurement sites revealed structures with dimensions of around 0.2 mm, which lies within the borderline range of image resolution of CBCT [11]. It is plausible that thin osseous lamellae might remain undetected by both MRI and CBCT methodologies. CBCT, although demonstrating good correlation with clinical measurements through transgingival probing [41], exhibited statistically significant deviations when compared to the gold-standard micro-CT [42]. Thus, CBCT presents an approximation of the true value, which could only be assessed by micro-CT or histological images that are beyond the scope of this clinical study.

Similar to an earlier study [40], the MRI acquisition protocol was successfully performed within a brief timeframe of less than 10 min, and no application of contrast agents was necessary. Dental MRI has been evaluated for preoperative diagnostic utility in dental implant placement [39], third molar surgery [43], and orthognathic surgery [40]. The employment of non-ionizing MRI offers the advantage of reduced radiation exposure when compared to CBCT, which is of particular concern in adolescents requiring follow-up examinations [44, 45]. Nevertheless, the clinical implementation of dental MRI remains constrained due to its limited accessibility, elevated costs [40], and its susceptibility to artefacts caused by metallic elements [46]. These considerations should be kept in mind by orthodontic practitioners prior to initiating orthodontic treatments if MRI investigations are planned. Another limitation of the dental MRI scans obtained in the current investigation is the absence of sufficient image contrast for accurately identifying the cemento-enamel junction (CEJ). This makes comparison of MRI with existing data obtained from CBCT studies difficult, as the CEJ often serves as a reliable landmark in radiographic images [23, 24, 37].

As all measurements and image reconstructions were performed by a single examiner, all measurements are similarly calibrated. However, this may also introduce a source of subjectivity. Although the image reconstruction and superimposition were done in a standardized manner by an experienced examiner, the post-processing of digital intraoral scans into finalized STL files can introduce deviations from the true gingival dimension. In addition, most software solutions require a scan repair to remove invalid elements or holes to ensure a more robust processing of the STL file during image registration, which in turn may alter the dimensions of the model.

In order to validate the results of the present study, future investigations conducted by multiple investigators are desirable. Finally, future studies might include larger sample sizes or the use of lip retractors for CBCT acquisition instead of superimposed intraoral scans.

## Conclusions

In the context of the current study, dental MRI and superimposed CBCTs effectively captured gingival dimensions within the buccal aspect of the anterior mandible, with clinically acceptable deviations.

## Data Availability

The data that support the findings of this study are not openly available due to reasons of sensitivity and are available from the corresponding author upon reasonable request.
